# Puerperal Fever; Its Treatment and Prevention

**Published:** 1883-01

**Authors:** John Lowe

**Affiliations:** Lichfield


PUERPERAL FEVER: ITS TREATMENT AND PREVENTION. By JOHN LOWE, L.R.C.S.I., L.R.C.P.E. and L.M., Lichfield.

No subject has created more anxiety or attracted more attention in recent years than that of puerperal fever; and as I have been unfortunate enough to have had some experience of this terrible disease, I do not wish to hide that experience, and the lessons derived from it, if its publication may prove useful to others.

I was engaged in a large London practice, nearly two years ago, where the number of confinements averaged over three hundred cases annually. Scarlet fever was very prevalent in the district, and later on erysipelas broke out as well. We isolated our cases most carefully, and were satisfied that a perfect system of disinfection was observed. We visited all the parturition cases earliest in the day, and I always used, and do so now, a mixture of carbolic acid and vaseline for aseptic purposes when attending confinements. I practiced, in fact, what is now called antiseptic midwifery. Notwithstading all this, a mild case of puerperal septicæmia occured in a case attended by my chief.

The abnormal tenderness, high but variable temperature, cessation of lochia, offensive stools, and lightheadedness, ending in maniacal behavior, combined to convince me that these were the expressive symptoms of puerperal fever poison, and Dr. Bristowe, who saw the case subsequently, fully concurred in this opinion. This case, after a protracted illness, eventually made a good recovery. A few days after the above case occured, the nature of the dire enemy was established beyond a doubt. Another woman, whom my principal had delivered of her second child, fell suddenly ill on the third day after delivery. She presented all the classic symptoms of malignant puerperal fever when I saw her twenty-four hours after the onset of the disease. I resorted to eliminate effete products from her blood, and had recourse to free stimulation and support. I cleared the lower bowel by enema, and I irrigated the uterus and neighboring pouches with carbolic acid solution every six hours. Fifty-two hours from the commencement of the disease pulmonary embolus manifested itself. It was now painful to witness the poor woman's suffering and it would be superfluous to describe it. She sank rapidly in the usual way. The third case, which was attended by myself, is more interesting, so I shall relate it more fully.

B. T., aged 27 years, had previous good health, but of bilious temperament. Delivered of third child on 28th Nov. 1880, with forceps and without difficulty. Did well until 5th Dec., when offensive diarrhœa began and lochia stopped. The temperature in axilla was 102.3° Fahr., and there were other minor symptoms. I gave her a strong powder containing calomel, jalap, and podophyllin, which improved the fæces in color and odor. Her diet was limited to broths and milk, with a little brandy occasionally. I washed out the uterus with carbolic acid solution, and the bladder with permanganate of potash solution, four times in twenty-four hours. On the 7th Dec. she fell into a thorough stupor, and could not be roused for more than two hours. After six hours she was semi-conscious, but her expression was very vacant, and her movements very purposeless. There was paralysis of left arm and leg, with constriction about pharynx, and inability to articulate. She had difficulty in swallowing liquids. Incontinence of urine and fæces—the former very ammoniacal, although bladder was regularly washed out. Pulse 112, temperature 104.5°. Pallor, loathing of food, thirst, full pulse, very contracted pupils, and intense pain in right temporal region, with paralysis—sensory and motor—aforesaid, clearly pointed to localized cerebritis, the result of septic thrombus. I prescribed half an ounce of brandy in milk to be given every hour. 8th.—Respiration an effort. Fæces offensive, and urine as before, with a specific gravity of 1029. Amblyopia, contracted pupils, and other symptoms persisting. Can speak a little. Ideation much impaired. Treatment continued, increasing brandy. Ordered her an ounce and a half of castor-oil. 9th.—Slight offensive discharge from vagina. Temperature very variable. Uterus and bladder washed out as before. Headache is still severe, but the fæces are less offensive and more normal in appearance. I have had ice applied to her head since I diagnosed cerebritis. 11th.—She had no proper sleep since the 5th inst. Milk has been gradually disappearing from her breasts, but there is none secreted now. Slight but less offensive uterine discharge. There has never been any pain about uterus or abdomen. Pupils less contracted. Sight imperfect. Headache persists. Urine and fæces improve. Treatment continued. 13th.—Less thirsty. Had an hour's good sleep last night. Looks vacant. Speaking a great toil. Movements very slow and deliberate. Fæces and urine improved. Cerebral macula on left hip. In other respects as before, and treatment continued. 16th.—Better in all respects, except that there is slight incontinence of urine. Appetite improved. Pupils act well. Sensation and motor power have returned in leg to a slight extent. She progressed slowly yet certainly afterwards, and when I last saw her on the 23rd she had enjoyed regular and natural sleep, relished her food, and said that she felt quite well, if it were not for the paralysis. She had perfect control over both bladder and rectum.

I have stated that I believe the cerebritis was caused by thrombus, and not by embolus, in this case, as, if the latter were afloat, then the lungs or liver would most likely suffer, as indeed they more frequently do. The treatment of septic cerebritis by administering brandy with easily digested but nutritious food, and the application of ice to the head, is suggested by common sense, and will be found useful in practice.

The fourth case was malignant in type occurring in a woman who was already in a hopeless condition from neglected syphilis. She exhibited the usual lung complication before death. The remaining five cases came under my notice early; and although each was in a very critical condition some time during the attack, yet they all recovered. One had periuterine inflammation for some time after, and another had uterine phlebitis with a large pyæmic abscess of the calf of one leg. I attribute recovery in these cases partly to their coming under treatment early, and partly to the good constitution of the women. In each case I encouraged free action of the liver, bowels, and kidneys, and I had frequent recourse to stimulation when it was indicated by past history or present symptoms.

I am strongly of opinion that by early and repeated aseptic intra-uterine injections, a rapidly acting cholagogue, washing out the bladder, if necessary, with some aseptic solution, and the timely and liberal use of stimulants, will avert death in many instances. It is no use giving the nurse instructions to wash out the uterus; we must do so ourselves by means of a long tube in the uterine cavity itself. Ammonia and brandy I regarded as the medicines for the disease; indeed when food is refused, brandy is not only most grateful to the patient, but it is peculiarly well adapted to supply the place of ordinary food, and no amount of fever or other symptom contra-indicates stimulation when changes so destructive to the vital fluids and tissues of the body are in terribly rapid progress. To give aconite or veratrum viride in such cases is, in my opinion, as unscientific as it is useless; and yet these remedies have been vaunted and are actually used by men of undoubted ability and eminence. To get rid of a fermentive poison from the blood we must adopt some such practice as I have indicated, and not stop to theorize about the physics of the circulation. We must, in other words, support vitality and eradicate the poison. That salicylates and sulpho-carbolates taken internally do not rectify the turbid urine in puerperal fever, I am convinced from experience; and I would strongly urge that all depressant remedies are both hurtful and dangerous. It would be a waste of time and space for me to speculate on the etiology of puerperal fever, still more to discuss the appropriate term for the disease. In the same run of cases we meet with malignant puerperal fever septicæmia, and pyæmia. For my part, I believe that either may give rise to the other, and that scarlet fever or erysipelas may undoubtedly cause any of the above forms of puerperal mischief. This is my experience, and I have watched more cases under the care of others than those referred to in this paper.

I have now to call attention to what would appear to have received least attention: how best to prevent contagion. The golden rule hitherto has been to leave off attending confinements altogether for several weeks. This is generally a safe rule, but it is often a mighty inconvenient, and, I believe a most unnecessary one. It occured to me that by the strictest asepticism the spread of the disease might be prevented. I remained as short a time as possible in the lying-in room when attending confinements, lubricated my hands well with vaseline and carbolic acid, and had the carbolic spray playing between the patient and me during operations. The labor over, I washed out the uterus and vagina with carbolic acid solution in the usual way. Now, this proceeding has the advantage of cleanliness and safety, and is unobjectionable. From the moment I took these precautions there was not another case of puerperal fever in the practice, although I continued to attend cases as they turned up, and I cannot see how any one can fail to appreciate the efficacy of the treatment pursued. The inconvenience of the spray and its formidable appearence are not valid arguments against the practice: one additional puerperal case would be more inconvenient and more formidable. I will not pursue this subject further now, but merely give the foregoing remarks—made with much sincerety and earnestness—to the profession for consideration.

---

PROF. W. T. MONTGOMERY intends leaving Chicago about Feb. 1, for a six months sojourn in Europe.

